# Gout Remission as a Goal of Urate-Lowering Therapy: A Critical Review

**DOI:** 10.3390/ph16060779

**Published:** 2023-05-23

**Authors:** Adwoa Dansoa Tabi-Amponsah, Sarah Stewart, Graham Hosie, Lisa K. Stamp, William J. Taylor, Nicola Dalbeth

**Affiliations:** 1 Department of Medicine, Faculty of Medical and Health Sciences, University of Auckland, Auckland 1023, New Zealand; dansoa.tabi-amponsah@auckland.ac.nz (A.D.T.-A.);; 2 School of Clinical Sciences, Faculty of Health and Environmental Sciences, Auckland University of Technology, Auckland 0627, New Zealand; sarah.stewart@aut.ac.nz; 3 Department of Medicine, University of Otago, Christchurch 8011, New Zealand; lisa.stamp@cdhb.health.nz; 4 Department of Medicine, University of Otago, Wellington 6242, New Zealand; william.taylor@otago.ac.nz

**Keywords:** gout, gout remission, preliminary gout remission criteria, urate-lowering therapy

## Abstract

Urate-lowering therapies for the management of gout lead to a reduction in serum urate levels, monosodium urate crystal deposition, and the clinical features of gout, including painful and disabling gout flares, chronic gouty arthritis, and tophi. Thus, disease remission is a potential goal of urate-lowering therapy. In 2016, preliminary gout remission criteria were developed by a large group of rheumatologists and researchers with expertise in gout. The preliminary gout remission criteria were defined as: serum urate < 0.36 mmol/L (6 mg/dL); an absence of gout flares; an absence of tophi; pain due to gout < 2 on a 0–10 scale; and a patient global assessment < 2 on a 0–10 scale over a 12-month period. In this critical review, we describe the development of the preliminary gout remission criteria, the properties of the preliminary gout remission criteria, and clinical studies of gout remission in people taking urate-lowering therapy. We also describe a future research agenda for gout remission.

## 1. Introduction

Gout is a chronic disease of monosodium urate (MSU) crystal deposition that presents clinically with intermittent gout flares, chronic gouty arthritis, and tophi [1]. Urate-lowering therapy leads to a reduction in serum urate levels and the dissolution of monosodium urate crystals. Ultimately, urate-lowering therapy also leads to the suppression of the clinical features of gout [2].

For many chronic rheumatic diseases, including rheumatoid arthritis, systemic lupus erythematosus, psoriatic arthritis, and idiopathic juvenile arthritis, remission is a key goal of treatment [3,4,5,6]. In chronic rheumatic diseases, remission has been defined as a “complete absence of disease activity or a level of disease activity so low that it is not troublesome to the patient and portends a later good prognosis” but has the possibility of relapse, i.e., “remission” is different from a “cure” [7]. With the availability of urate-lowering therapy, remission should be considered a treatment goal for people with gout. However, there are complexities to defining remission in gout, particularly the disease course which involves intermittent gout flares with long inter-critical periods without symptoms. Some older studies used the term “remission” to describe an absence of gout flares and/or a decrease in serum urate levels [8,9,10]. However, until recently, there has been no agreed definition of gout remission. In 2016, de Lautour et al. [11] described preliminary gout remission criteria based on the following OMERACT-endorsed core outcome domains for long-term gout studies: serum urate, gout flares, tophi, patient global assessment, and pain due to gout [12].

The objective of this critical review is to summarise research that has defined gout remission and outline a future research agenda. Articles were identified using electronic databases (PubMed/Medline, EMBASE and Cochrane). Search terms included “gout” AND “remission” OR “complete response” OR “low disease activity”. Searches were filtered to include publications between 2016 and 2023 to ensure that the articles identified were published after the development of the preliminary gout remission criteria. The references of relevant articles were also screened.

## 2. Development of Preliminary Gout Remission Criteria

**Figure 1:**
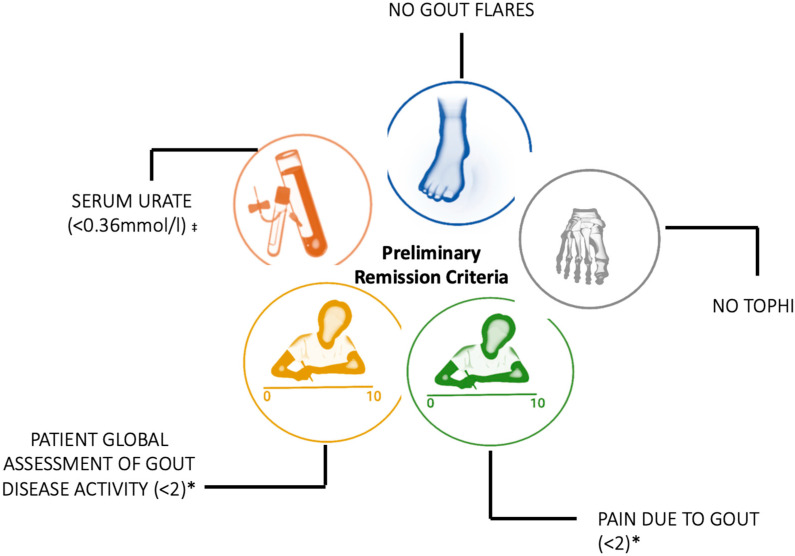
**Preliminary gout remission criteria.** All domains must be fulfilled over a 12-month period to achieve gout remission. ‡ Measured at least twice at equal distances apart over a 12-month period, all values must be <0.36 mmol/L. * Measured at least twice at equal distances apart over a 12-month period on a 0–10-point scale, no values ≥ 2. Figure produced by ADT-A.

Preliminary gout remission criteria were developed in 2016 using the Delphi consensus and 1000 Minds discrete choice methodology with 49 participants who were clinicians and researchers with gout expertise. The preliminary gout remission criteria were eventually defined as: serum urate < 0.36 mmol/L (6 mg/dL); an absence of gout flares; an absence of tophi; pain due to gout < 2 on a 0–10 scale; and a patient global assessment < 2 on a 0–10 scale (Figure 1). All criteria must be met for a person to be considered in gout remission.

There were several areas of discussion and uncertainty as the criteria were developed. Participants raised concerns about the specificity of the pain and patient global assessment criteria and whether other health conditions could influence the scoring of these patient-reported outcome domains [11,13]. This discussion led to an agreement that the pain domain would be assessed using a questionnaire that asked about “pain due to gout”. The wording of the patient global assessment questionnaire was also changed to “considering all of the ways in which your gout affects you, how well have you been doing in the last week?” [11].

Considerations of the fluctuating course of gout led to the agreement that serum urate, pain, and the patient global assessment should be assessed at least twice over a specific timeframe. The timeframe over which remission would be measured was a challenging issue, and agreement through the Delphi process could not be reached between 6 months or 12 months. Discussions about these two timeframes highlighted trade-offs between feasibility and validity. In research settings, assessing remission over a 6-month period would reduce cost and resource. However, remission over a 12-month period would offer stronger evidence of absent disease activity. In a 1000 Minds exercise assessing different timeframes, the 12-month period was preferred over 6 months [11].

Since the publication of these preliminary gout remission criteria, studies have analysed the properties and validity of these criteria, the proportion of people with gout able to achieve gout remission on urate-lowering therapy, and predictors of gout remission in the setting of urate-lowering therapy.

## 3. Properties of the Preliminary Gout Remission Criteria

Table 1 summarises the studies that have examined the properties of the preliminary gout remission criteria and how the criteria relate to selected measures of gout disease activity. The table also includes clinical trials that reported on the proportion of people fulfilling and maintaining the criteria. These studies will be discussed in the following text.

The construct validity of the preliminary gout remission criteria was assessed by Dalbeth et al. [14] using dual-energy computed tomography (DECT), an advanced imaging technique that allows for the visualisation and quantification of MSU crystal deposition. The participants in this study were on at least 300 mg of allopurinol daily, with monitored recruitment for approximately 25% of participants with palpable tophi and approximately 50% with serum urate < 0.36 mmol/L (6 mg/dL). Fewer participants who met the preliminary gout remission criteria had MSU crystal deposition on the DECT (44%) than those who did not achieve the remission criteria (73.6%) (*p* = 0.004). The median DECT urate crystal volume was 0.00 (0.00–0.46) cm^3^ for those in remission, compared to 0.08 (0.00–19.53) cm^3^ in those not achieving remission (*p* = 0.002). With the exception of the pain domain, participants fulfilling the individual domains of the remission criteria had lower volumes of DECT-detected urate crystal deposition. The DECT-detected urate crystal volume was independently associated with the serum urate domain and the tophus domain of the preliminary gout remission criteria.

The preliminary gout remission criteria also align with other disease activity assessments in patients receiving urate-lowering therapy. In the DECT study of patients on allopurinol, patients and physicians completed questionnaires rating their assessment of current gout control using a numerical rating scale (0 = not at all controlled; 10 = fully controlled) [15]. Participants who fulfilled the preliminary gout remission criteria reported higher gout control scores than those who did not fulfil these criteria. Similarly, physicians rated higher gout control scores for participants who fulfilled the preliminary gout remission criteria [15].

A Gout Activity Score (GAS_3c_) has been developed and validated by Scirè et al. [16]. The GAS includes similar domains to the gout remission criteria and is derived from the following formula:

$$\begin{array}{c} 0.09\times \mathrm{last}\;12\;\mathrm{month}\;\mathrm{gout}\;\mathrm{flares}+1.01\times \sqrt{\mathrm{serum}\;\mathrm{urate}}\\ \;\;\;\;\;\;\;+0.3\times \mathrm{patient}\;\mathrm{global}\;\mathrm{assessment}\;\left(\mathrm{visual}\;\mathrm{analogue}\;\mathrm{scale}\right)\\ \;\;\;\;\;\;\;+0.53\times \ln\left(1+\mathrm{tophi}\;\mathrm{number}\right) \end{array}$$

The authors used the preliminary gout remission criteria to determine the best cut-off associated with the lowest misclassification of gout remission and consider a GAS < 2.5 as gout remission.

Schlesinger et al. [17] developed a “complete response” criteria for gout in the setting of assessing outcomes of people with very severe gout who were treated with pegloticase (an infusion agent that leads to almost undetectable plasma urate levels in treatment responders) and reported “complete response” and remission in the pegloticase responders. The authors argued that a statistically derived “complete response” approach may be preferable for a remission construct in this setting. This was partly due to their observation that not all domains of the remission criteria correlated or improved in the same way within the study population in response to pegloticase. This finding may not be a limitation of the remission criteria (and indeed may indicate a strength of a composite measure that captures different aspects of disease). The proposed “complete response” definition included serum urate, tophus, patient global assessment, swollen joint count, and tender joint count but not gout flares or pain. In the 32 pegloticase responders in this open-label extension study, 23 (71.9%) met the “complete response” criteria and 27 (84.4%) fulfilled the preliminary gout remission criteria.

## 4. Can the Preliminary Gout Remission Criteria Be Achieved on Urate-Lowering Therapy?

### 4.1. Fulfilment of Preliminary Gout Remission Criteria

In the DECT study by Dalbeth et al. [14], the preliminary gout remission criteria, assessed cross-sectionally, were fulfilled in 23 (15.1%) of the 152 participants who were on 300 mg or more of daily allopurinol. Of note, this study population may not reflect a typical allopurinol-treated population due to the study design, which included monitored recruitment for approximately 25% of participants with palpable tophi and only 50% with serum urate < 0.36 mmol/L (6 mg/dL).

The study by Schlesinger et al. [17], reported the fulfilment of the preliminary gout remission criteria in pegloticase-treated participants from two parallel randomised controlled trials and their open label extension (OLE) studies [20,21]. Of the 56 participants who entered the OLE and had been on 8 mg biweekly infusions of pegloticase, 27 (48.2%) fulfilled the preliminary gout remission criteria. These participants had been on the pegloticase treatment for 6 months, and pegloticase responders were more likely to fulfil the criteria; of the 32 responders, 27 (84.4%) fulfilled the preliminary gout remission criteria. In contrast, only 8.3% of the non-responders achieved gout remission even when the requirement for serum urate < 0.36 mmol/L was omitted.

In a 5-year longitudinal study of 500 people with gout on an oral urate-lowering therapy (primarily with allopurinol at doses up to 750 mg daily), Alvarado-de la Barrera et al. [18] reported the fulfilment of the preliminary gout remission criteria. After 1 year of follow-up, 9.1% of participants had fulfilled the criteria. This was followed by 30%, 28% 39%, and 28% at Years 2, 3, 4, and 5 respectively. None of the participants with severe gout at baseline—defined as ≥5 tophi and/or intradermal tophi— fulfilled the preliminary gout remission criteria over the five-year period.

In another longitudinal study, Cipolletta et al. [19] recruited 50 participants who had received either allopurinol or febuxostat for at least 6 months. Participants who fulfilled all domains of the preliminary gout remission criteria at baseline were included in the study, and over the 12-month follow-up, 42% maintained remission using the preliminary gout remission criteria. There was no difference in remission maintenance between allopurinol or febuxostat users.

### 4.2. Fulfilment of the Serum Urate Domain

Consistent with their study recruitment plan, Dalbeth et al. [14] found that 51% of participants fulfilled the serum urate domain. The proportion of participants fulfilling the serum urate domain was not reported by Schlesinger et al. [17]; however, the 32 pegloticase responders were defined as having a persistent lowering of serum urate below 0.36 mmol/L (6 mg/dL) for greater than or equal to 80% of the time during months 3 and 6 of the two parallel randomised controlled trials. Alvarado-de la Barrera et al. [18] did not report the proportion of participants fulfilling this domain; however, in the non-severe gout group, a target urate of <0.36 mmol/L (6 mg/dL) was achieved in 50–70% of participants followed for 3–5 years. In the severe gout group, fewer than 50% of participants achieved a target urate of <0.30 mmol/L (5 mg/dL). In the Cipolletta et al. [19] observational study, 72.4% of participants maintained the serum urate domain over the 12 months.

### 4.3. Fulfilment of the Gout Flares Domain

Dalbeth et al. [14] reported that 46.1% of participants fulfilled the gout flare domain. The proportion of participants fulfilling this domain was not reported by Schlesinger et al. [17] or Alvarado-de la Barrera et al. [18]. In the Cipolletta et al. [19] study, 66% of participants maintained this domain over 12 months.

### 4.4. Fulfilment of the Tophus Domain

Consistent with the study recruitment plan, the tophus domain was fulfilled by 68.4% of participants in the study by Dalbeth et al. [14]. Schlesinger et al. [17] did not report the proportion of participants fulfilling this domain. In the Alvarado-de la Barrera et al. [18] study, none of the participants with severe gout, defined as ≥5 tophi and/or intradermal tophi at the baseline visit, fulfilled the preliminary gout remission criteria, and the main reason for this was persistent tophi. All the participants in the Cipolletta et al. [19] study maintained this domain over 12 months.

### 4.5. Fulfilment of the Pain and Patient Global Assessment Domains

In the Dalbeth et al. [14] study, 68.4% of participants fulfilled the pain domain, and 55.2% fulfilled the patient global assessment domain. The proportion of participants fulfilling these domains was not reported by Schlesinger et al. [17] or Alvarado-de la Barrera al. [18]. Cipolletta et al. [19] reported that 55.2% of participants maintained the pain domain and 48.3% maintained the patient global assessment domain over 12 months.

## 5. Can We Predict Achievement of Gout Remission during Urate-Lowering Therapy?

The available studies indicate that for people with gout, remission as defined by the preliminary gout remission criteria is achievable on urate-lowering therapy. Identifying predictors of remission could offer valuable insight into how gout management could be improved so that there is equitable achievement of gout remission across different clinical and demographic groups.

A key finding from Alvarado-de la Barrera et al. [18] was that patients with high tophus burdens at baseline did not achieve remission according to the preliminary gout remission criteria, even on high doses of allopurinol for five years. For these participants, the target serum urate was difficult to achieve. Participants with high tophus burdens also had longer disease durations, lower socioeconomic and educational levels, and more comorbidities. These findings support the earlier use of urate-lowering therapy to prevent the consequences of severe gout and greater support for people with lower socioeconomic and educational levels or those with complex co-morbid health conditions.

Beyond these clinical and demographic variables, imaging studies may also be useful in predicting which patients are likely to achieve and maintain gout remission on urate-lowering therapy. Cipolletta et al. [19] demonstrated that baseline ultrasound estimation of the MSU crystal burden was an independent predictor of gout remission maintenance over one year of urate-lowering therapy. In this study, the MSU crystal burden was assessed via ultrasound scanning of the elbow, wrist, hand, knee, ankle, and foot for aggregates, the double contour sign, and tophi. The ultrasound findings were then combined to produce a total ultrasound score for each participant. In those participants without ultrasound evidence of MSU crystal deposition, 87.5% maintained gout remission compared to 66.7% of those with evidence of crystal deposition (*p* < 0.01). The authors calculated that over the 12-month study, for each 1-point increase in the total ultrasound score, the risk of not maintaining the preliminary gout remission criteria increased 1.81-fold. Together with the DECT data [14], this study indicates that people with gout who have lower baseline urate crystal burdens on advanced imaging have a greater chance of achieving and maintaining gout remission as defined by the preliminary gout remission criteria.

In addition to imaging biomarkers, laboratory biomarkers that can predict the fulfilment of gout remission also need to be evaluated. Laboratory biomarkers such as serum CA72-4, which has been identified as a predictor of gout flares, could be useful in predicting gout remission [22]. Metabolic proteins in the pathogenesis of gout, such as those involved in purine metabolism, branched amino acid metabolism, and proteins in the complement and coagulation pathways, could also prove to be effective predictors [23,24]. Although there are studies of circulating laboratory biomarkers as potential predictors of gout disease activity [25,26], these studies have not used the preliminary gout remission criteria as outcome measures. Analyses of serum and plasma samples obtained from clinical trials and longitudinal observational studies will be beneficial for the identification and validation of biomarkers that can predict the achievement of gout remission.

## 6. Gout Remission as a Goal of Urate-Lowering Therapy—What Are the Next Steps?

Questions remain about whether all the domains of the preliminary gout remission criteria are necessary to define gout remission, which treatment strategies lead to high rates of gout remission, and whether achieving and maintaining gout remission leads to other clinical benefits.

### 6.1. Are All the Domains of the Criteria Necessary to Define Gout Remission?

Of the five domains that contribute to the preliminary gout remission criteria, serum urate is the most consistently measured primary outcome in studies of urate-lowering therapy [27]. In published clinical trials, there is an underrepresentation of other OMERACT core outcome domains that are also required to report remission outcomes; 80% report serum urate, 70% report gout flare, 10% report tophus, 17% report pain, and 7% report patient global assessment [27]. Therefore, most trials have not measured all the domains required to assess remission according to the preliminary gout remission criteria.

It is possible that some domains within the preliminary gout remission criteria are overlapping—in other words, they may be measuring the same construct—and that gout remission could be defined using fewer domains. In particular, gout flares, pain due to gout, and patient global assessment of gout may overlap. Dalbeth et al. [14] and Schlesinger et al. [17] have considered the overlap or association between the individual remission domains in their respective studies. In an analysis of the individual domains, Dalbeth et al. [14] noted that the highest overlap was found between the pain and patient global assessment domains (50.7%). Overlap between the pain domain, patient global assessment, and gout flares domain was also suggested by Schlesinger et al. [17]; pain and patient global assessment were worse at the time of a gout flare, and were lower during periods of no gout flares [17].

The OMERACT gout core outcome domains were developed with patient research partners; however, to date, patient perspectives on gout remission have not been reported. As we continue to investigate gout remission as a goal of urate-lowering therapy, patient perspectives will be crucial to ensuring that these criteria represent outcomes that are relevant to people with gout. This work may also allow for the understanding of whether there is duplication or overlap in the individual domains when considering the state of gout remission.

### 6.2. What Treatment Strategies Lead to Gout Remission?

To date, studies have demonstrated that gout remission, as defined by the preliminary gout remission criteria, can be achieved in some people with gout who receive the urate-lowering medications allopurinol, febuxostat, and pegloticase. Additional comparative efficacy analyses, both in clinical trials and in real-world healthcare settings, will be useful for understanding whether gout remission rates are higher with different urate-lowering therapies. This will require consistent data collection not only for serum urate but also for the other remission domains (gout flares, tophus, pain, and patient global assessment) which are less frequently measured and reported [27].

In addition to comparing specific urate-lowering therapy medications, there is also scope to compare gout remission outcomes across different methods of the health care delivery of urate-lowering therapies. There has been increasing interest in the role of different models of care for gout management, including pharmacist-led care, nurse-led care, and multidisciplinary team care [28]. In a large clinical trial, nurse-led gout care was more effective than the usual GP-led care in reducing serum urate, reducing gout flares, and improving tophus outcomes over two years [29]. To date, gout remission rates have not been reported as an outcome in studies examining outcomes of different models of gout care.

A further issue when considering urate-lowering therapy is the role of anti-inflammatory prophylaxis (typically with low-dose colchicine or NSAIDs) at the time of initiating the urate-lowering therapy. This approach has the potential to address both the MSU crystal deposition and the inflammatory components of gout, both of which are captured by the preliminary gout remission criteria. While anti-inflammatory prophylaxis has been shown to reduce the frequency of gout flares during the first 3–6 months of treatment [30,31], it is unknown whether anti-inflammatory prophylaxis has additional benefits in the proportion of patients achieving gout remission or time to achieve gout remission.

### 6.3. Does Gout Remission Lead to Improvement in Other Important Outcomes?

A key aspect of the concept of remission is prognosis, specifically that being in the state of remission “portends a later good prognosis” [7]. When considering gout prognosis, this might include better physical function and health-related quality of life (HRQoL), less radiographic damage, or fewer comorbid health conditions. To date, it is not known whether being in the state of gout remission leads to improvement in these outcomes. This question will require an analysis of clinical trial data or longitudinal studies that have measured all the domains within the preliminary gout remission criteria (serum urate, gout flares, tophus, pain due to gout, and patient global assessment) and other outcome measures of function, HRQoL, radiographic damage, or incident comorbid conditions over at least 24 months.

## 7. Conclusions

For people with gout on urate-lowering therapy, remission can be achieved and should be a goal of therapy. Preliminary gout remission criteria have been developed by rheumatologists and researchers with expertise in gout to allow for the clinical assessment and reporting of gout remission. These criteria capture a range of domains, reflecting both MSU crystal deposition and the inflammation induced by MSU crystals. These criteria have undergone some validation and have been used to describe the proportion of participants achieving gout remission in longitudinal studies. Several questions remain about the definition of gout remission, particularly whether the definition fully captures the patient experience, whether all domains are required, and whether being in remission leads to improvements in other important outcomes for people with gout. In addition, it is currently unknown how best to achieve gout remission and whether we can predict remission in people starting urate-lowering therapy. Answering these questions will require consistent measurement of the OMERACT core outcome domains and other health outcomes in long-term gout studies.

## Figures and Tables

**Table 1:** Studies that examined the properties of the preliminary gout remission criteria and how the criteria relate to other measures of gout disease activity as well as studies that reported the proportion of people fulfilling and maintaining the criteria.

Research Article	Objective	Disease Activity Measures	Study Population	Urate-Lowering Therapies
**“Concurrent validity of provisional remission criteria for gout: a dual-energy CT study” Dalbeth et al., 2019** [14]	To test the concurrent validity of the preliminary gout remission criteria by comparing the criteria with dual energy CT (DECT) findings.	Dual-energy CT urate crystal volume. Preliminary gout remission criteria.	People with gout on allopurinol ≥ 300 mg daily. Enrolment included at least 25% of participants with subcutaneous tophi and 50% with serum urate < 0.36 mmol/L (6 mg/dL)	Of participants, 84.6% had been on 300 mg allopurinol daily for at least 3 months. Of participants,18.4% had been on >300 mg allopurinol daily for at least 3 months.
**“Predictors of patient and physician assessment of gout control”Dalbeth et al., 2022** [15]	To understand the clinical variables that contribute to the patient and physician assessment of gout control.	Patient gout control score. Physician gout control score. Preliminary gout remission criteria.	People with gout on allopurinol ≥ 300 mg daily. Enrolment included at least 25% of participants with subcutaneous tophi and 50% with serum urate < 0.36 mmol/L (6 mg/dL).	Of participants, 84.6% had been on 300 mg allopurinol daily for at least 3 months. Of participants, 18.4% had been on >300 mg allopurinol daily for at least 3 months.
**“Development and First Validation of a Disease Activity Score for Gout”Scirè et al., 2016** [16]	To develop a new composite disease activity score for gout and provide its first validation.	Composite disease activity score which included the following: o Serum urate concentration. o Gout flares in last 12 months. o Patient global assessment (0–10 visual analogue scale). o Tophi number. Preliminary gout remission criteria.	People with a clinical diagnosis of gout referred to 30 rheumatology clinics across Italy as part of the Kick-Off of the Italian Network for Gout [KING] study.	Of participants, 68.7% had been on allopurinol. Of participants, 13.6% had been on febuxostat.
**“Evaluation of proposed criteria for remission and evidence-based development of criteria for complete response in patients with chronic refractory gout.”Schlesinger et al., 2019** [17]	To define criteria for complete response in people with chronic refractory gout treated with pegloticase and to determine the proportion of patients with chronic refractory gout treated with pegloticase who meet the preliminary remission criteria.	Complete response criteria which included the following: o Serum urate <0.36 mmol/L (6 mg/dL). o Resolution of all tophi. o Patient global assessment ≤1. o Swollen joint count ≤1. o Tender joint count ≤1. Preliminary gout remission criteria.	Fifty-six people with chronic refractory gout defined as baseline serum urate ≥ 0.48 mmol/L (≥8 mg/dL), and one or more of the following: o Three or more self-reported gout flares during the previous 18 months. o One or more tophi. o Gouty arthropathy defined clinically or radiographically as joint damage caused by gout. o Contraindication to treatment with allopurinol or history of failure to normalize serum urate despite maximally appropriate allopurinol dose.	Biweekly intravenous infusions of 8 mg pegloticase for 6 months.
**“Are target urate and remission possible in severe gout? A 5-year cohort study.”Alvarado-de la Barrera et al., 2020** [18]	To determine the proportion of patients achieving target serum urate, defined as less than <6 mg/dL (0.36 mmol/L) for patients with non-severe gout and <5 mg/dL (0.30 mmol/L) for patients with severe gout, and to determine the proportion of participants fulfilling the preliminary gout remission criteria.	Preliminary gout remission criteria.	A total of 221 people with severe gout (≥5 tophi at baseline visit) and 279 participants with non-severe gout.	Allopurinol up to 750 mg daily (95% of participants). Probenecid (1.4–7.5% of participants)—dosing strategy not described.
**“Sonographic estimation of monosodium urate burden predicts the fulfilment of the 2016 remission criteria for gout: a 12-month study”Cipolletta et al., 2021** [19]	To investigate whether baseline monosodium urate crystal burden estimated by ultrasound predicts achievement of gout remission.	Preliminary gout remission criteria.	Fifty people with gout recruited from inpatient and outpatient clinics who satisfied all domains of the preliminary remission criteria at baseline.	Of participants, 68% had been on allopurinol for at least 6 months—dosing strategy not described. Of participants, 32% had been on febuxostat for at least 6 months—dosing strategy not described.

Though different candidate Gout Activity Scores (GAS) were developed, this table only includes the 4-variable GAS which appeared as the best candidate.

## Data Availability

Not applicable.
